# Molecular Characterization of Trichophyton Species by Arbitrarily Primed (AP)-PCR: A Genotypic Differentiation Approach

**DOI:** 10.7759/cureus.116087

**Published:** 2026-09-11

**Authors:** Sheetal Sharma, Bhawani Shankar Verma, Kuldeep Yadav, Uzma Rahman, Sadaf Rahman, Akansha Puri, Sandeep Dogra

**Affiliations:** 1 Microbiology, Government Medical College and Hospital, Jammu, Jammu, IND; 2 Microbiology, Prince Medical College and Hospital, Sikar, IND; 3 Microbiology, Marudhara Medical College and Hospital, Jodhpur, IND; 4 Biotechnology, Chandigarh University, Chandigarh, IND

**Keywords:** dermatophyte identification, microsporum, molecular diagnostics, superficial mycoses, trichophyton, trichophyton species

## Abstract

Background

Dermatophytes are keratinophilic fungi affecting the skin, hair, and nails. Conventional identification based on culture and morphology may be limited in differentiating closely related species. Therefore, this study aimed to evaluate the molecular profiling and genotypic discrimination of *Trichophyton *species using arbitrarily primed (AP)-PCR.

Materials and methods

Four common dermatophyte species were specifically detected and differentiated in the current investigation using AP-PCR: *Trichophyton rubrum*, *Trichophyton mentagrophytes*, *Trichophyton tonsurans*, and *Trichophyton verrucosum*. Four different arbitrary primers were used to amplify the genomic DNA of these species: OPAA11 (5' ACCCGACCTG 3'), OPU15 (5' ACGGGCCAGT 3'), OPAA17 (5' GAGCCCG ACT 3'), and OPD18 (5' GAGAGCCAAC 3').

Results

Of the 242 superficial dermatophyte infections examined, 173 were positive for *Trichophyton* species. Four species were identified: *T. mentagrophytes* (98 isolates), *T. rubrum* (58 isolates), *T. tonsurans* (12 isolates), and *T. verrucosum *(five isolates). *T. mentagrophytes* was the predominant species, whereas *T. rubrum* predominated among nail infections. AP-PCR confirmed and differentiated the four *Trichophyton* species at the molecular level.

Conclusion

By overcoming the consistency and specificity constraints of traditional phenotypic approaches, AP-PCR provides a quick, dependable, sensitive, and discriminatory molecular methodology for precise dermatophyte detection.

## Introduction

Dermatophytes are keratinophilic fungi responsible for superficial mycoses (tinea/ringworm) of the skin, hair, and nails. They belong mainly to three genera: *Epidermophyton*, *Microsporum*, and *Trichophyton*. *Epidermophyton* contains two species, while *Microsporum* mainly infects skin and hair. *Trichophyton* is the most diverse genus and can infect skin, hair, and nails, with important human pathogens including *Trichophyton mentagrophytes*, *Trichophyton rubrum*, *Trichophyton tonsurans*, and *Trichophyton verrucosum* [1-2].

*Trichophyton* species have a wide variety of colonial morphologies, with surfaces varying from farinose-like to silky or glossy. A key morphological requirement for species-specific recognition within the genus is this macroscopic diversity combined with distinctive reverse coloration. Every species of *Trichophyton* dermatophyte has distinctive patterns of pigment synthesis and colonial design. Microconidia and macroconidia, respectively, are seen in all *Trichophyton* species when examined under a microscope. Additionally, several *Trichophyton* species exhibit particular auxotrophy characteristics related to nourishment. *T. mentagrophytes* requires methionine for optimal growth, whereas *T. tonsurans* shows a dietary requirement for the amino acids ornithine, citrulline, and arginine. These nutritional requirements may assist in species identification using selective diagnostic media [2-3]. In vitro culture techniques and microscopic examination are the two principal methods used in laboratory studies for dermatophytosis.

Microscopy offers a rapid preliminary detection of fungal elements but depends on observer expertise and generally lacks species-level specificity. Culture remains the standard for definitive dermatophytosis diagnosis, using clinical specimens grown on mycological media and identified by characteristic microscopic and macroscopic morphology. However, culture is more specific but time-consuming, often requiring several days to weeks for fungal growth [4].

Rapid and accurate identification of dermatophyte species is essential in clinical mycology. Molecular methods, particularly arbitrarily primed (AP)-PCR, provide a rapid and reliable approach for species identification by generating species-specific DNA polymorphisms. AP-PCR is a PCR-based molecular biology technique used to compare DNA patterns between organisms or samples. These distinct banding patterns observed after agarose gel electrophoresis enable differentiation of dermatophyte species and offer advantages over conventional diagnostic methods [5-6].

Molecular methods offer faster and more sensitive dermatophyte detection than conventional techniques by targeting stable genetic characteristics [3,5]. The current study used AP-PCR to identify *Trichophyton* species in dermatophyte-infected patients. AP-PCR was combined with conventional phenotypic and microscopic methods to provide accurate and comprehensive identification.

## Materials and methods

Ethical approval

This study was conducted in the Post Graduate Department of Microbiology, Government Medical College and Hospital, Jammu, India. The study was approved by the Institutional Ethics Committee of GMC Jammu (IEC/GMCJ/2023/1485). 

The samples were collected from patients suffering from skin infections who visited the Outpatient Department of Government Medical College and Hospital, Jammu. All cases were enrolled after obtaining written informed consent in their vernacular language.

Study population and sample collection

The study included 320 patients with clinically suspected superficial dermatophytosis, comprising 272 patients with skin lesions and 48 with onychomycosis. Demographic and clinical data, including age, sex, occupation, duration of infection, previous infections, and relevant comorbidities, were recorded through standardized interviews. Previous antifungal treatment before sample collection was also documented.

The afflicted anatomical area was meticulously treated using a 70% ethanol solution prior to specimen capture. After that, specimens were gathered using either a specialized sterilized rotary or a standard nail clipper (for nail samples) or a sterile, blunt-edged surgical blade (for skin lesions), and they were promptly placed into sterilized tubes. Following collection, each of the samples was divided into three separate portions: one for mycological culture, one for a standard microscopic inspection, and one for nucleic acid extraction.

Direct microscopic examination

The obtained samples were examined under a microscope after being cleared with a 20% potassium hydroxide (KOH) solution. A microscope was then used to view specimen slides under ×40 and ×100 (oil immersion) objectives. The detection of fungal hyphae, spores, and budding cells was the main focus of the thorough inspection.

Culturing

To stop bacterial proliferation, samples obtained were seeded onto Sabouraud Dextrose Agar (SDA) amended with gentamicin (20 mg/mL). After that, cultures were incubated for seven to 15 days at 37°C. Upon observing fungal development, colony morphology - which includes traits including size, configuration, uniformity, and coloration on both the obverse and reverse sides of the Petri plate - was used for macroscopic identification. Lactophenol cotton blue tease mounts were then used for microscopic analysis. The unique structural traits of the hyphae, fruiting bodies, and spores of fungal cultures were used to identify them [4-6]. 

DNA extraction

Fungal DNA was systematically extracted by mechanically disrupting the specimens in the presence of proteinase K, lysis buffer, and 10% sodium dodecyl sulfate (SDS), followed by incubation at 65°C for two to three hours. The samples were vortexed, mixed with cetyltrimethylammonium bromide (CTAB)/NaCl, and incubated at 56°C for one hour. An equal volume of chloroform (24:1) was then added, and the samples were centrifuged at 7000 rpm for 10 minutes at 25°C. The supernatant was transferred to fresh tubes and treated with an equal volume of phenol:chloroform (25:24:1). The samples were subsequently centrifuged at 7000 rpm for 10 minutes, and the resulting supernatant was transferred to fresh tubes. An equal volume of cold isopropanol was added, and the samples were incubated at -20°C for 30 minutes to precipitate the DNA. Following centrifugation at 7000 rpm for 10 minutes at 4°C, the DNA pellet was washed three times with 70% ethanol, air-dried, and reconstituted in 50 µL of sterile Milli-Q water. The extracted DNA was stored at -20°C until further molecular analysis [5].

AP-PCR

Each primer was individually used for AP-PCR of all isolates. Amplification reactions were prepared in 20 µL total volume that included 3 µL of DNA template, 10 µL of master mix, 2 µL of one out of four random primers [OPAA11 (5’ - ACCCGACCTG-3’), OPU15 (5’ - ACGGGCCAGT-3’), OPAA17 (5’- GAGCCCGACT-3’) or OPD18(5’- GAGAGCCAAC-3’)], and 5 µL sterile Milli-Q water.

Thermocycling was carried out at the following settings: 32 cycles of denaturation (94°C for 30 seconds), primer annealing (36°C for 45 seconds), and extension (72°C for 90 seconds). Post-PCR products were separated through electrophoresis on a 1.2% agarose gel in 1× Tris-acetate-EDTA (TAE) buffer. The gel was stained with ethidium bromide using a Bio-Rad Gel Doc EZ Imager (Bio-Rad Laboratories, Hercules, CA) [5,7]. 

Sequencing

Internal transcribed spacer (ITS) region amplification was used to further analyze the purified PCR amplicons in order to definitively identify dermatophytes. DNA Baser application (Heracle BioSoft, Mioveni, Romania) was used to perform bioinformatic assessment on the sequences of nucleic acids that were obtained from both forward and reverse cycle sequencing processes. This approach comprised sequence pruning and eventual alignment with existing reviewed sequence information accessible in the GenBank repository via the National Center for Biotechnology Information (NCBI) Basic Local Alignment Search Tool (BLAST) [6,8].

## Results

A total of 242 cases of superficial dermatophyte infections were examined. Among the identified isolates, *T. mentagrophytes* was recovered from 98 cases (40.5%), including 52 males and 46 females, followed by *T. rubrum* from 58 cases (24.0%; 37 males and 21 females), *T. tonsurans* from 12 cases (5.0%; four males and eight females), and *T. verrucosum* from five cases (2.1%; three males and two females). The remaining 69 cases (28.5%) were attributed to other dermatophyte species and/or isolates that were not included in the present species-specific analysis. Five clinical forms of dermatophytosis were recorded: tinea faciei, tinea pedis, tinea unguium (onychomycosis), tinea corporis, and tinea cruris. Among the 98 *T. mentagrophytes* isolates, 52 (53.1%) were obtained from tinea corporis, 34 (34.7%) from tinea cruris, seven (7.1%) from tinea faciei, and five (5.1%) from tinea pedis. Among the 58 *T. rubrum* isolates, 15 (25.9%) were obtained from tinea corporis, 41 (70.7%) from tinea cruris, and two (3.4%) from tinea pedis. *T. tonsurans* was identified in eight (66.7%) cases of tinea corporis and four (33.3%) cases of tinea cruris, whereas *T. verrucosum* was isolated from four (80.0%) cases of tinea corporis and one (20.0%) case of tinea faciei.

Within the species-specific groups, tinea corporis accounted for 53.1% (52/98) of *T. mentagrophytes* infections, followed by tinea cruris at 34.7% (34/98), tinea faciei at 7.1% (7/98), and tinea pedis at 5.1% (5/98). For *T. rubrum*, tinea cruris accounted for 70.7% (41/58) of cases, followed by tinea corporis at 25.9% (15/58) and tinea pedis at 3.4% (2/58). All 12 *T. tonsurans* isolates were distributed between tinea corporis (66.7%; 8/12) and tinea cruris (33.3%; 4/12). Among the five *T. verrucosum* isolates, 80.0% (4/5) were associated with tinea corporis and 20.0% (1/5) with tinea faciei. In this study, 173 samples positive for *Trichophyton* dermatophytes were examined using fungal culture and KOH microscopy. Genomic DNA was extracted from the culture isolates, and AP-PCR was employed for species-level identification. Four *Trichophyton* species were identified: *T. mentagrophytes* (98; 56.6%), *T. rubrum* (58; 33.5%), *T. tonsurans* (12; 6.9%), and *T. verrucosum* (five; 2.9%). Their identification was subsequently confirmed by AP-PCR (Table 1; Figures 1-2).

**Table 1:** Trichophyton species and primers with their corresponding molecular weight (band length) ranges. OPAA11, OPU15, OPAA17, and OPD18 represent the primers used for molecular characterization of the different *Trichophyton* species. The values indicate the observed molecular weight ranges of amplified DNA bands, expressed in base pairs (bp), for each primer-species combination.

	Primers and range of molecular weight in base pair			
Dermatophyte species	OPAA11	OPU15	OPAA17	OPD18
Trichophyton mentagrophytes	350-750	250-600	300-900	350-1000
Trichophyton rubrum	350-650	250-800	300-1200	350-1300
Trichophyton tonsurans	350-850	250-900	300-1200	350-1400
Trichophyton verrucosum	350-600	250-750	300-800	350-1100

**Figure 1:**
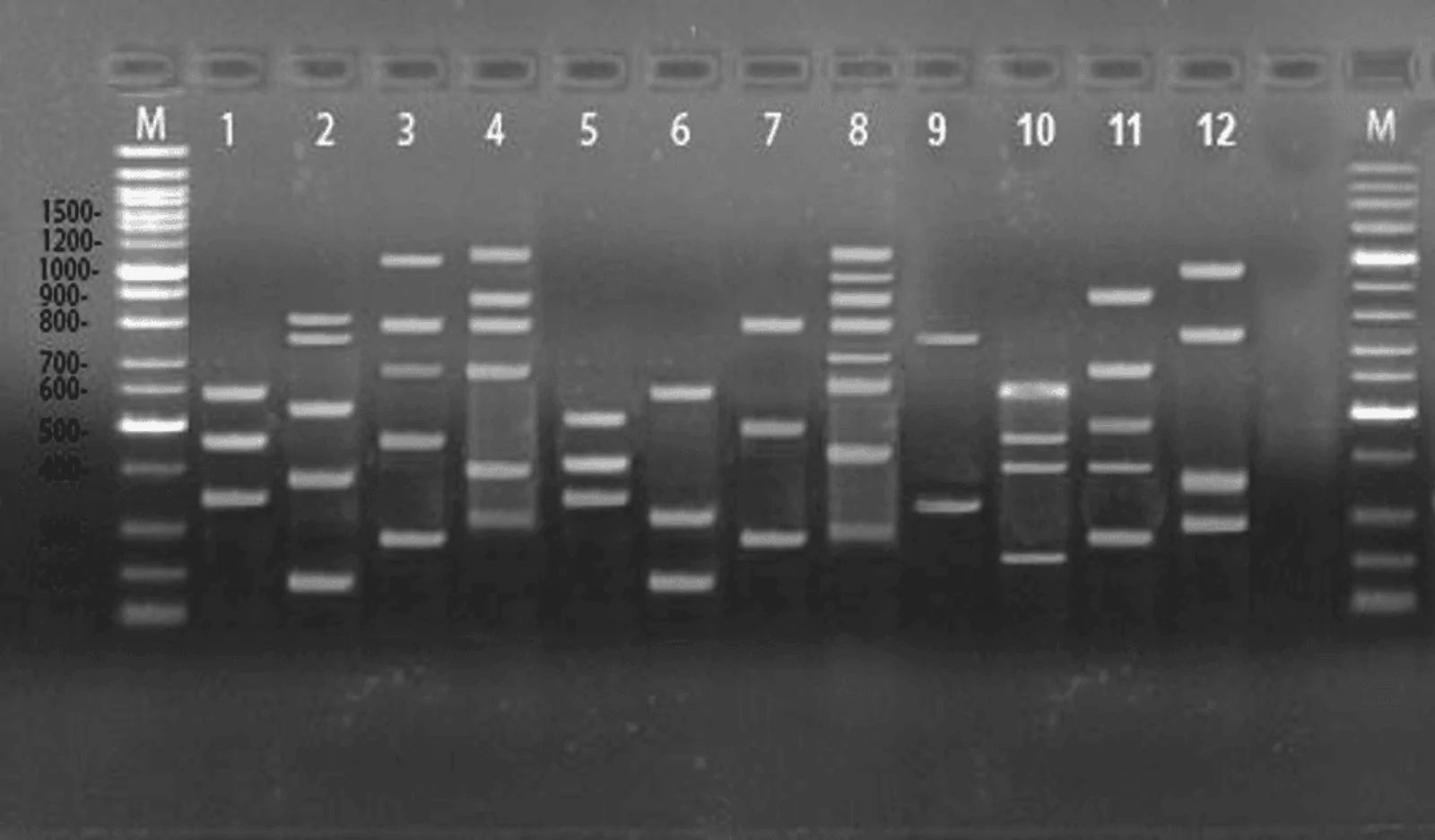
The genomic DNA of Trichophyton rubrum, Trichophyton verrucosum, and Trichophyton mentagrophytes was amplified using primers OPAA11, OPU15, OPAA17, and OPD18. The results of primers OPAA11, OPU15, OPAA17, and OPD18 derived from *Trichophyton rubrum* are shown in rows 1-4. The results of primers OPAA11, OPU15, OPAA17, and OPD18 from *Trichophyton verrucosum* are seen in lanes 5-8. Lanes 9-12 show the primers of *Trichophyton mentagrophytes*.

**Figure 2:**
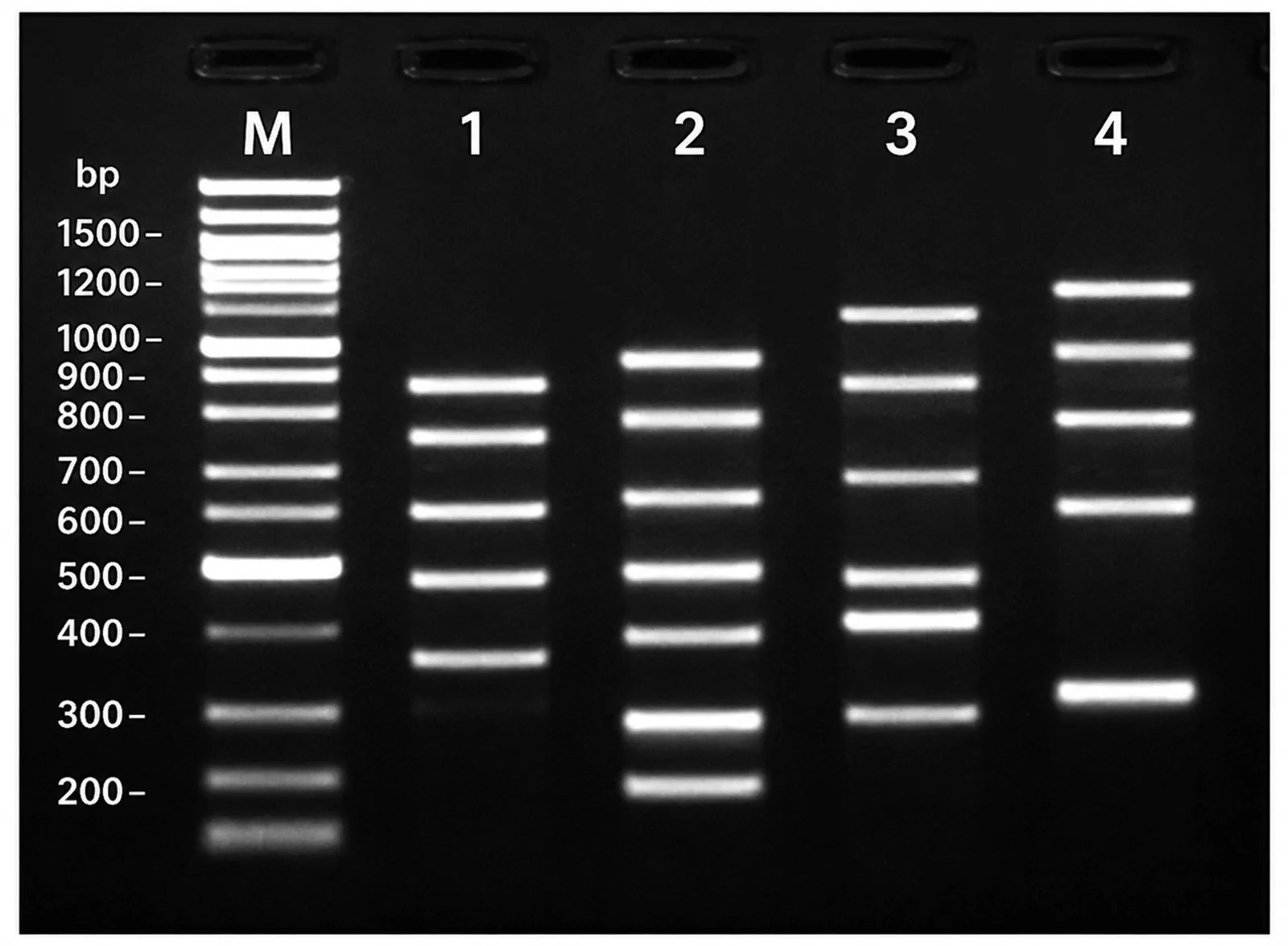
Amplified products of genomic DNA of Trichophyton tonsurans obtained from primers OPAA11, OPU15, OPAA17 and OPD18. Lanes 1-4 show the products of primers OPAA11, OPU15, OPAA17, and OPD18 from *Trichophyton tonsurans*, respectively.

After six to 10 days of incubation, *T. mentagrophytes* typically produces well-developed colonies on SDA. The colonies may vary from powdery or fluffy to white, creamy, or downy, often with characteristic pigmentation on the reverse. Microscopically, the species exhibits septate hyphae and characteristic grape-like clusters of microconidia (Figure 3).

**Figure 3:**
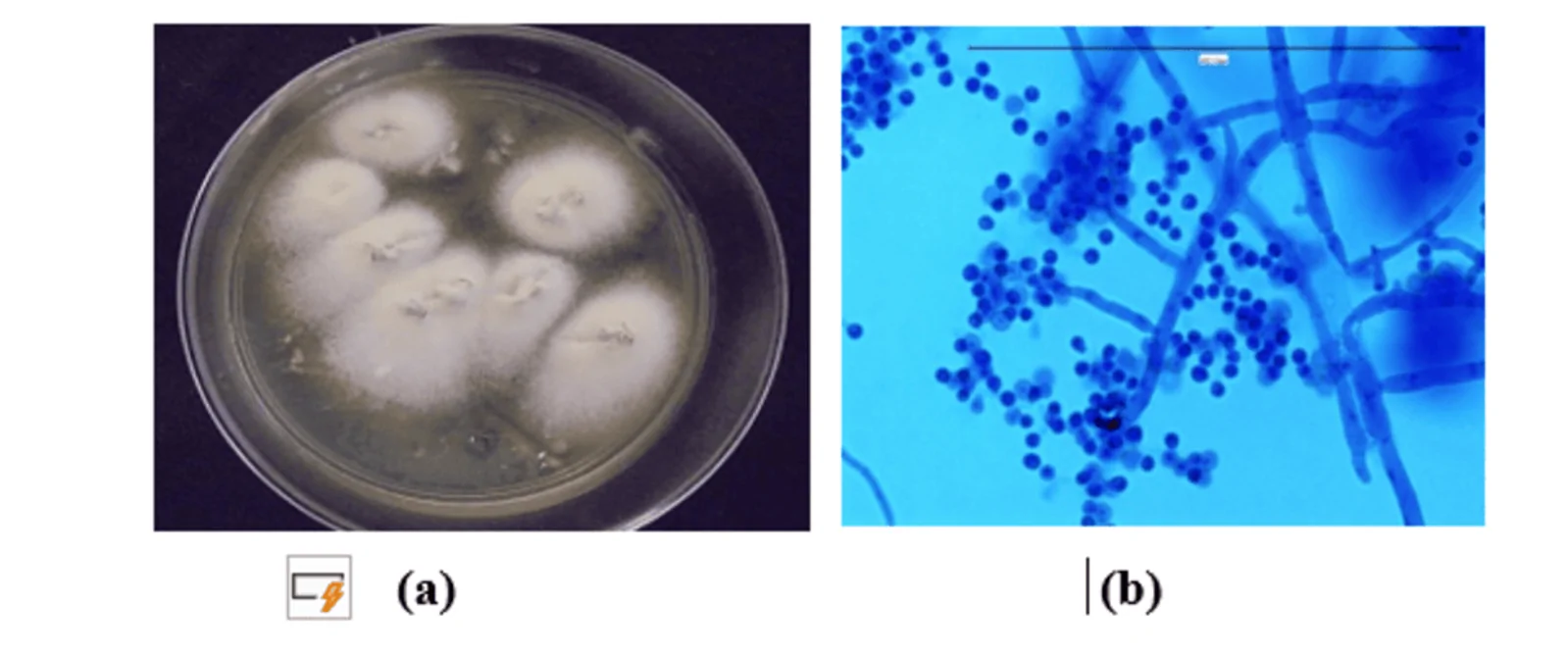
(a) Colonies of Trichophyton mentagrophytes on Sabouraud Dextrose Agar (SDA) media at 37°C up to seven days of growth. (b) Microscopic image of microconidia (40×).

Compared with other dermatophyte species, *T. rubrum* exhibits relatively slow growth. On culture media, it typically produces white, smooth to woolly colonies, with a characteristic reddish-brown pigmentation on the reverse side of the colony. Microscopically, numerous teardrop-shaped microconidia are observed, whereas macroconidia are rarely present. The characteristic cultural and microscopic features of *T. rubrum* are shown in Figure 4.

**Figure 4:**
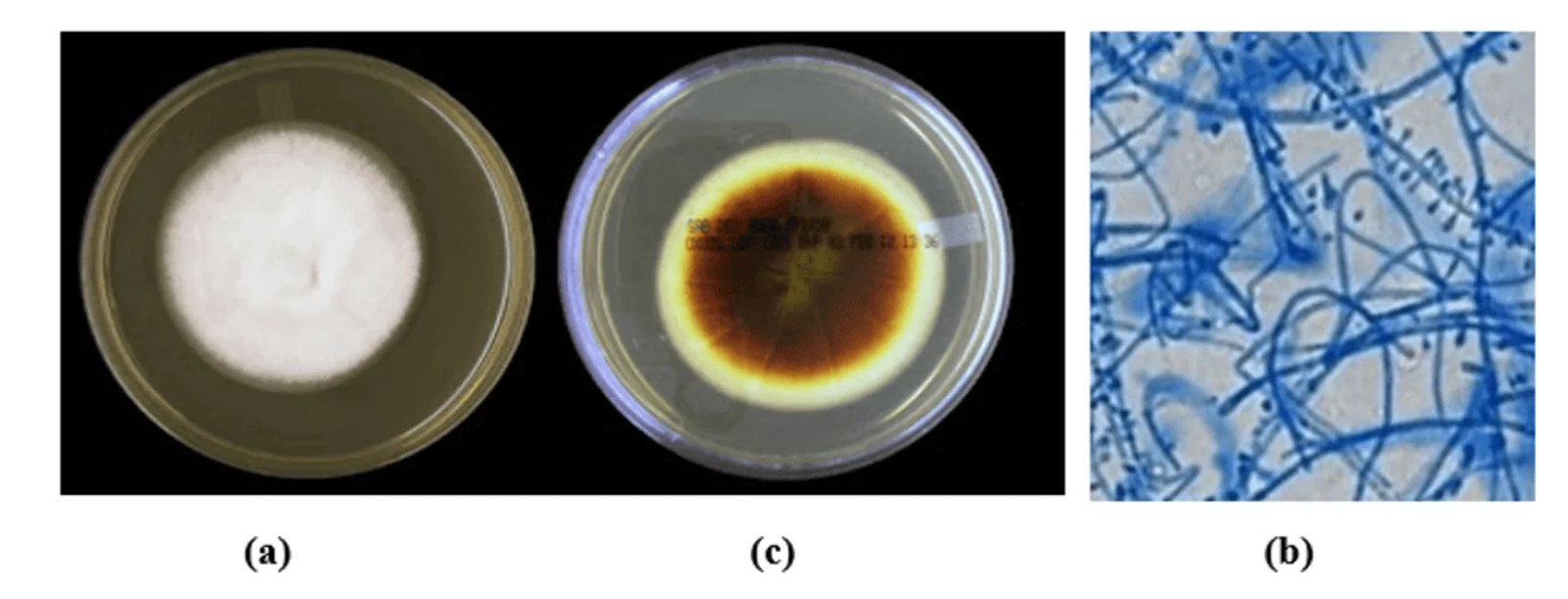
(a) Colonies of Trichophyton rubrum on Sabouraud Dextrose Agar (SDA) media at 37°C up to seven days of growth. (b) Reverse side of colonies on media plate. (c) Microscopic image of microconidia (40×).

In our observations, *T. rubrum* exhibited relatively rapid growth, producing flat, firm, pale-white colonies that were characteristically folded at the center. Microscopically, macroconidia were rarely observed, whereas microconidia were abundant and showed considerable morphological variation. They typically had a broad base and appeared in teardrop-shaped, cylindrical, or balloon-like forms, as shown in Figure 5.

**Figure 5:**
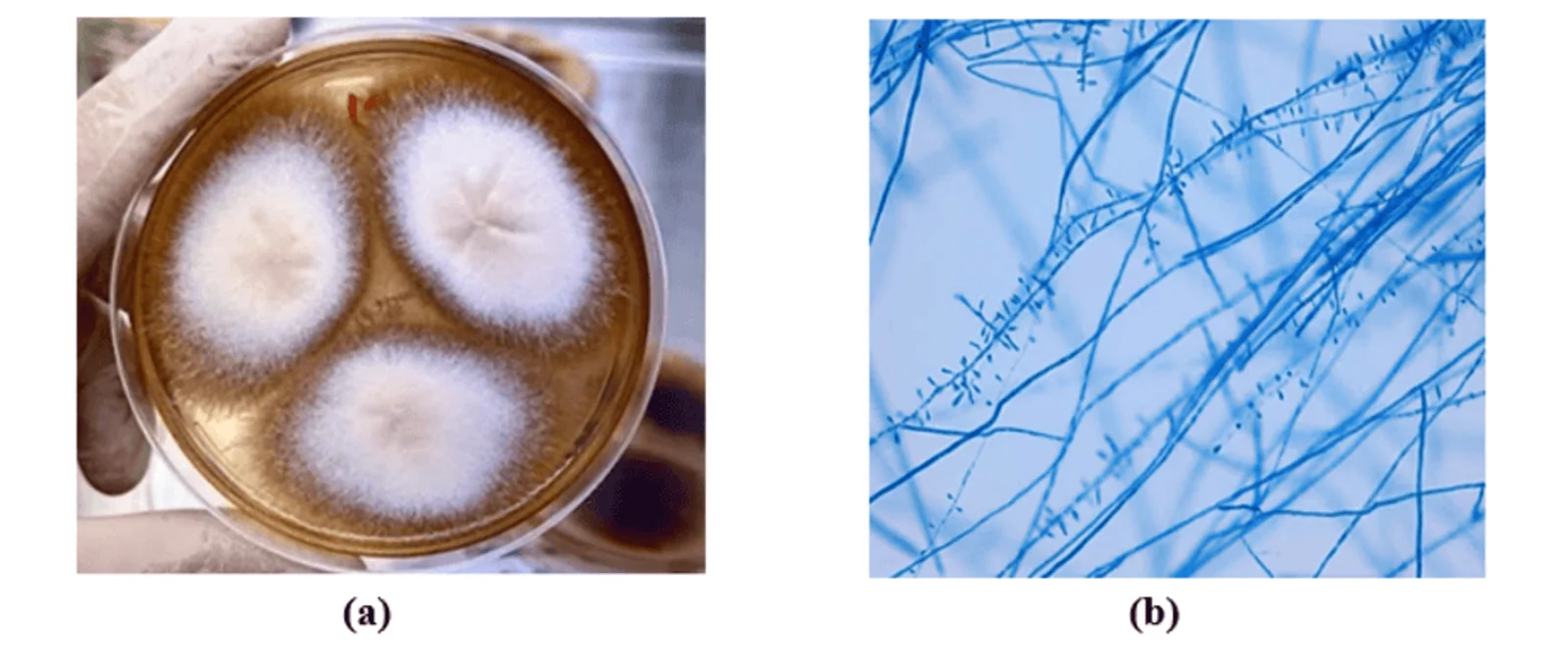
(a) Colonies of Trichophyton tonsurans on Sabouraud Dextrose Agar (SDA) media at 37°C up to seven days of growth. (b) Microscopic image of microconidia under (40×).

*T. verrucosum*, in contrast, exhibits extremely slow growth. Macroscopically, it produces white, flat to creamy-colored colonies on culture media. Microscopically, it is characterized by tear-shaped microconidia and distinctive string-bean-shaped macroconidia. The characteristic cultural and microscopic features of *T. verrucosum* are shown in Figure 6.

**Figure 6:**
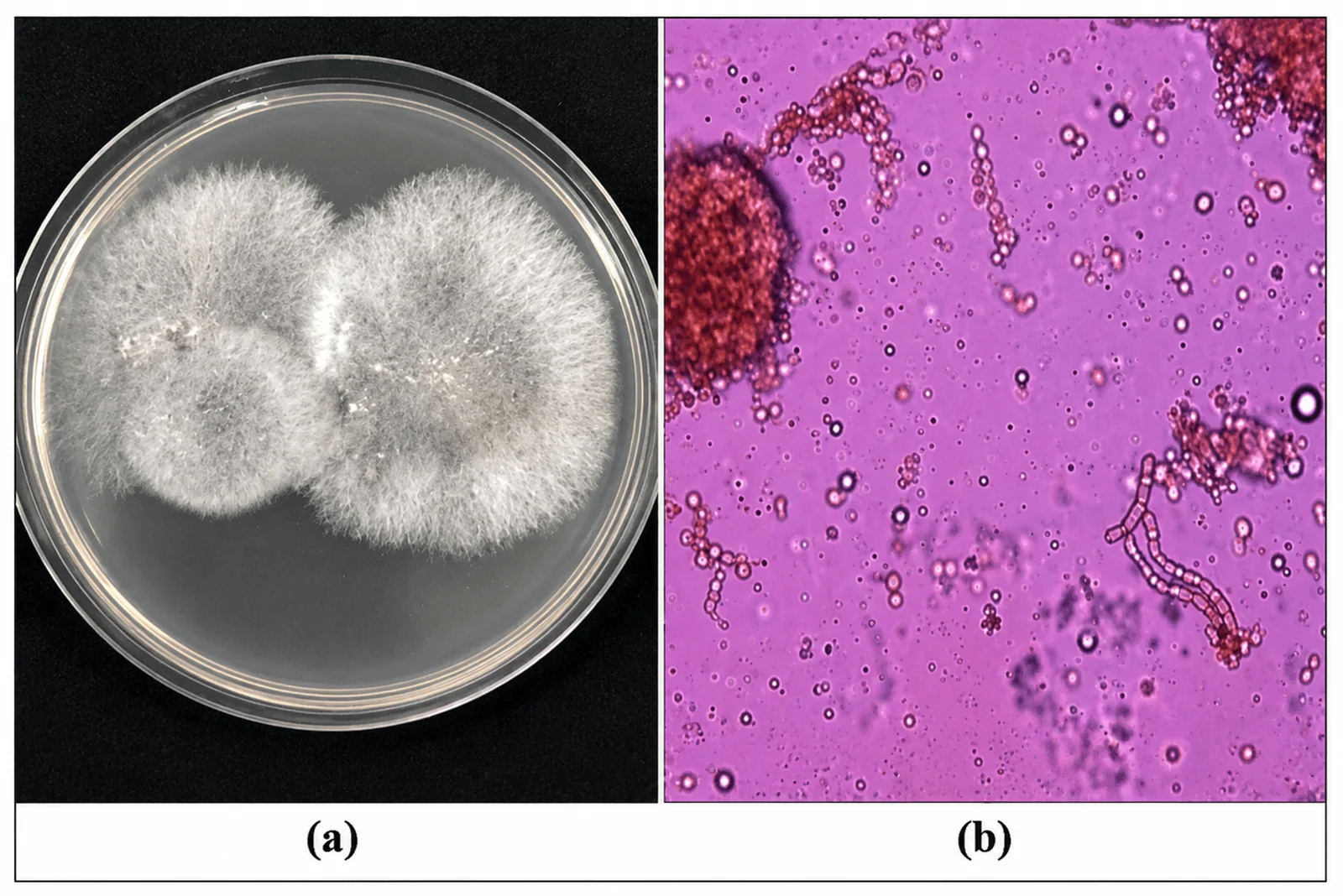
(a) Colonies of Trichophyton verrucosum on Sabouraud Dextrose Agar (SDA) media at 37°C up to seven days of growth. (b) Microscopic image of microconidia (40×).

Four *Trichophyton* species - *T. mentagrophytes*, *T. rubrum*, *T. tonsurans*, and *T. verrucosum* - were identified using AP-PCR with the random primers OPAA11, OPU15, and OPD18 (Figures 1-2). Seven dermatophyte strains that were obtained from people with tinea cruris and tinea corporis had their ITS regions transcribed in the current study. The NCBI BLAST algorithm was used to identify the standard DNA sequences that were extracted from the PCR results and amplified utilizing the ITS1 and ITS4 primer combination.

## Discussion

AP-PCR was used to identify dermatophytes from skin and nail specimens and was evaluated against conventional KOH microscopy and culture methods. Using four arbitrary primers - OPAA11, OPU15, OPAA17, and OPD18 - AP-PCR proved to be a selective, economical, and reliable method for species identification. These findings are consistent with previous studies reporting successful dermatophyte detection using similar arbitrary primers, including OPAA17, either alone or in combination with other primers [8-9]. Although conventional methods are useful, dermatophyte identification is laborious and time-consuming. In contrast, AP-PCR provides rapid and specific species identification, with results obtainable within a day. Direct microscopy is rapid but lacks sufficient specificity for reliable species-level identification [4,7,10]. Seven isolates of fungal species were found in this investigation utilizing both traditional and microscopic techniques. These isolates included members of the genera *Trichophyton*, *Epidermophyton*, and *Microsporum*. The most common isolates discovered in skin and hair tissue specimens were *Trichophyton* species. These results are consistent with research done in Australia [1,3], where *Trichophyton* species were shown to be the most common dermatophytes linked to dermatophytosis. However, additional research from Iraq revealed that the most frequent isolates were *Microsporum* species, subsequently followed by *Trichophyton* species [6,11]. The observed variation in the prevalence of dermatophyte species may be attributed to geographical distribution and genetic diversity among strains. In the present study, seven dermatophyte isolates were successfully identified by AP-PCR using four random primers OPAA11, OPU15, OPAA17, and OPD18, with *Trichophyton* species being the most frequently isolated. All primers successfully amplified DNA, producing band patterns ranging from approximately 250 to 1400 bp, consistent with previous studies demonstrating their effectiveness in molecular identification of dermatophytes [7,9,12]. The isolates that were first identified as *T. mentagrophytes* were further identified as *T. mentagrophytes* and *Trichophyton interdigitale*, or *T. mentagrophytes* var. *interdigitale*, by decoding the amplification fragments. For dermatophyte determination, the ITS1 and/or ITS4 sections have been amplified and then sequenced. Numerous sequence alignments have confirmed the existence of particular "barcode" sequences in the ITS1 and/or ITS4 sections of different dermatophyte species, allowing for accurate molecular differentiation [13-14].

## Conclusions

The present study demonstrates the feasibility of using AP-PCR for molecular profiling and genotypic discrimination of the *Trichophyton* isolates examined. The primers OPAA11, OPU15, OPAA17, and OPD18 generated distinct amplification profiles that enabled differentiation among the four *Trichophyton* species investigated. These findings support the potential utility of AP-PCR as a molecular profiling approach; however, further studies with larger sample sizes and appropriate comparative analyses are required to determine its diagnostic performance and clinical utility.
